# Short stay hospital admissions for an acutely unwell child: A qualitative study of outcomes that matter to parents and professionals

**DOI:** 10.1371/journal.pone.0278777

**Published:** 2022-12-16

**Authors:** Cari Malcolm, Emma King, Emma France, Richard G. Kyle, Simita Kumar, Smita Dick, Philip Wilson, Lorna Aucott, Stephen W. Turner, Pat Hoddinott

**Affiliations:** 1 School of Health and Social Care, Edinburgh Napier University, Edinburgh, United Kingdom; 2 Nursing, Midwifery and Allied Health Professions Research Unit, University of Stirling, Stirling, United Kingdom; 3 Academy of Nursing, College of Medicine and Health, University of Exeter, Exeter, United Kingdom; 4 Screening and Immunisation, Public Health Scotland, Edinburgh, United Kingdom; 5 Child Health, University of Aberdeen, Aberdeen, United Kingdom; 6 Centre for Rural Health, University of Aberdeen, Aberdeen, United Kingdom; 7 Centre for Randomised Healthcare Trials, University of Aberdeen, Aberdeen, United Kingdom; 8 NHS Grampian, Aberdeen, United Kingdom; Sunway University, MALAYSIA

## Abstract

**Background:**

Numbers of urgent short stay admissions (SSAs) of children to UK hospitals are rising rapidly. This paper reports on experiences of SSAs from the perspective of parents accessing urgent care for their acutely unwell child and of health professionals referring, caring for, or admitting children.

**Methods:**

A qualitative interview study was conducted by a multi-disciplinary team with patient and public involvement (PPI) to explore contextual factors relating to SSAs and better understand pre-hospital urgent care pathways. Purposive sampling of Health Board areas in Scotland, health professionals with experience of paediatric urgent care pathways and parents with experience of a SSA for their acutely unwell child was undertaken to ensure maximal variation in characteristics such as deprivation, urban-rural and hospital structure. Interviews took place between Dec 2019 and Mar 2021 and thematic framework analysis was applied.

**Results:**

Twenty-one parents and forty-eight health professionals were interviewed. In the context of an urgent SSA, the themes were centred around shared outcomes of care that matter. The main outcome which was common to both parents and health professionals was the importance of preserving the child’s safety. Additional shared outcomes by parents and health professionals were a desire to reduce worries and uncertainty about the illness trajectory, and provide reassurance with sufficient time, space and personnel to undertake a period of skilled observation to assess and manage the acutely unwell child. Parents wanted easy access to urgent care and, preferably, with input from paediatric-trained staff. Healthcare professionals considered that it was important to reduce the number of children admitted to hospital where safe and appropriate to do so.

**Conclusions:**

The shared outcomes of care between parents and health professionals emphasises the potential merit of adopting a partnership approach in identifying, developing and testing interventions to improve the acceptability, safety, efficiency, and cost-effectiveness of urgent care pathways between home and hospital.

## Introduction

Epidemiological data show a persistent rise in the rate of unscheduled short stay hospital admissions of children across Scotland [1, 2] and the wider United Kingdom (UK) [3–6]. This increase in admissions is particularly striking amongst infants [5, 7, 8] and for conditions such as upper respiratory infection, viral infection, tonsillitis, bronchiolitis, and lower respiratory infection [1–5]. In addition to rises in unscheduled short stay admissions (SSAs), paediatric attendances at emergency departments (ED) are also increasing in the UK, with the resultant impacts of ED over-crowding, long waiting times, and an increased burden to health services. Paediatric ED attendance rose from 3.9 million to 5.1 million in the six years to 2015, and as many as 60% of these presentations were for non-urgent reasons [9]. During the SARS-CoV-19 pandemic, urgent healthcare use by children reduced [10, 11] but live monitoring of admissions and ED presentations in Scotland shows presentations are at or above activity in 2018–2019 [12]

Factors contributing to the increased number of emergency department (ED) attendances and SSAs of children, where the admissions typically last for no more than 24 hours, are varied and complex. They include, but are not limited to, changes in parents’ health seeking behaviours and expectations of the health service; parental worry or overestimation of the severity of their child’s illness; challenges in accessing or navigating unscheduled and community-based care services; a trend towards shorter duration of hospital stay with potential for increased readmissions; and the introduction of UK policy changes such as the maximum four-hour waiting time target for assessment in hospital EDs, primary care reforms and opening of paediatric short stay assessment units [3–5, 8, 9, 13].

It is widely acknowledged by health professionals, service managers and policy makers that continuing this trajectory of rising ED attendances and SSAs puts additional pressures on an already overstretched health service and is not sustainable [4, 9, 14]. Therefore evidence-based interventions are required which aim to ensure the right care is provided in the right place at the right time from the perspective of families and service providers.

The FLAMINGO project (**FL**ow of **A**d**M**issions in ch**I**ldren and you**NG** pe**O**ple) is a mixed-methods study consisting of three sequential phases designed to provide novel insights into the pathway leading to urgent SSAs in children. Phase one, using linkage of national datasets, examined the pre-referral pathways for and characteristics of paediatric SSAs. All paediatric hospital medical admissions during the calendar years of 2015 and 2017 in Scotland were linked to databases for the following referral sources: ED; general practice (GP); out of hours general practice outside working hours (OOH) and NHS24, which includes the Scottish Ambulance Service. Detailed methods and results of the data linkage are reported separately [15]. Phase two was informed by the data analysed in phase one and involved undertaking qualitative interviews with both parents and health professionals to explore contextual factors relating to SSAs, better understand referral pathways, and develop priorities for future interventions aimed at improving unscheduled care pathways and the appropriateness of SSAs. In phase three, an engagement event attended by phase two participants and wider stakeholders was held to share project findings and debate and prioritise interventions identified during the interviews.

The aim of this paper is to report findings from phase two of the FLAMINGO study and provide an in-depth account of the experiences of SSAs from the perspective of parents accessing unscheduled urgent care for their acutely unwell child and of health professionals referring, caring for, or admitting children for SSAs.

## Methods

### Study design

A qualitative exploratory approach was used to gain a better understanding of the experiences of parents and health professionals with regards to the circumstances around unscheduled SSAs of children to hospital. The term ‘children’ has been used throughout this paper with the understanding that it includes infants, children, and young people (up to the age of 16). Reporting adhered to the Consolidated Criteria for Reporting Qualitative Studies (COREQ) guidelines [16] (See S1 File).

### Study setting

The FLAMINGO study was undertaken in Scotland, where the National Health Service (NHS) is organised into 14 geographically distinct terrestrial Health Boards, each responsible for healthcare provision to their region’s population. Apart from the three Health Boards that have few or no inpatient paediatric facilities (NHS Orkney, Shetland, and Western Isles), data from the remaining 11 regional Health Boards were included in the data linkage undertaken in phase one [15]. For phase two, the FLAMINGO project team purposively identified five Health Boards to act as cases that would allow for maximal variation in characteristics such as deprivation, urban-rural, and hospital structure (with or without a dedicated children’s hospital or short stay facility). Case selection was also informed by the quantitative data emerging from the data linkage exercise to allow for variation in numbers of unscheduled hospital admissions.

### Participants and sampling

Purposive sampling was used to invite medical and nursing health professionals working in the five case Health Boards, with experience of paediatric urgent care pathways, and responsibility for referring or receiving children for acute paediatric medical admission. Sampling took place across primary care, out of hours services (OOH), and hospital ED/PEDs workplaces. Potential participants were emailed an information sheet about the study, an invitation to participate, and contact details for the main researcher (EK) to arrange a telephone interview.

Parents were eligible to participate if they had experience of a SSA for their child (16 years of age and younger) in one of the five case Health Boards. A SSA was defined as admission to hospital under the care of inpatient paediatric services typically for a period of less than 24 hours. However, for the two areas of the country with a PED, a SSA would also include those occasions where children are seen and then discharged directly from the PED. The SSA must have been for an acute medical illness and have taken place within the past five years. SSAs for reasons other than acute medical illness, such as for surgical conditions were excluded.

The COVID-19 pandemic coincided with our qualitative recruitment phase and associated restrictions such as ‘lockdowns’ meant that previously planned recruitment methods could not be carried out. It was our original intention to recruit parents to the study in public areas including hospital foyers and shopping centres in our Health Board sites. Alternative convenience sampling methods were adopted including online platforms and social media, press releases and advertisements in local newspapers. Similar to the approach used for inviting health professionals, parents responding to the invitation were sent a participant information sheet, study details and guidance on how to contact the main researcher (EK) to arrange a telephone interview. Due to the challenges of recruiting to health research during the pandemic [17], combined with the project’s objective of ensuring maximal variation of participants to be able to meet the aims of the study, the final recruitment press release, disseminated in January 2021, accepted potentially information-rich participants from outside the five Health Board case areas providing they met the other inclusion criteria. In addition, Phase 1 quantitative analysis and early interviews with families raised seizures as a reason for an urgent short stay admission that is worthy of further study. Towards the end of the qualitative data collection, further purposive samples of health professionals and families with experience of seizure were recruited and mixed method findings will be reported separately.

### Patient and Public Involvement (PPI)

Patient and Public Involvement was a central tenet of the FLAMINGO project, as advocated by leading UK research funders and organisations [18, 19]. PPI was established at the outset of the project to ensure the views of children and parents were considered at all stages. Engagement with parents and stakeholders, through attendance at nine parent-toddler groups and one university-based PPI advisory group, enabled them to share their experiences of accessing healthcare and attending hospital, to inform the development of the project’s interview topic guide and ensure the materials used for recruitment, such as leaflets and posters, were appropriate. Initial PPI discussions revealed that many parents were unsure at what point in a hospital visit that their child was officially categorised as a SSA. The quantitative analysis of the data linkage analysed SSA where admission and discharge occur on the same calendar date [15]. To improve clarity when recruiting parents for interview, the definition of a SSA was therefore adapted to include stays in hospital lasting less than 24 hours. A PPI advisor critically reviewed and commented on drafts of this manuscript.

### Data collection

Semi-structured interviews were chosen as they would allow for consistency between the two interviewers (EK, CM) whilst still allowing interviewees to raise issues of importance. Separate semi-structured interview topic guides were developed for parents and health professional interviews by our multidisciplinary research team, with input from PPI and drawing on existing literature on urgent short stay hospital admissions (See S2 and S3 Files).

The interviews took place between December 2019 and March 2021, with a three month pause in recruitment due to the COVID-19 lockdown between March and May 2020. COVID-19 had dramatic effects on hospital admissions and access to health care, with the changes in behaviour across society impacting on the prevalence of childhood infections [10]. Original plans to conduct face-to-face interviews were altered and instead were conducted via telephone. The COVID-19 pandemic was a context disruptor and data collected during this period might not be fully representative of typical SSA practices; therefore, interviewees were asked to speak about experiences pre-COVID. Interviewees were then asked specifically if and how they had observed differences during, or as a consequence of, the pandemic. Interviewees were assured that confidentiality would be ensured and their anonymity protected.

Interviews were conducted by two experienced qualitative researchers (EK, CM) with different backgrounds (health services research and paediatric nursing) and reflective field notes were kept. The duration of the interviews ranged from 18 to 62 minutes (median 27) and each interview was audio recorded, anonymised and then transcribed verbatim.

### Data analysis

Anonymised transcripts were uploaded to QSR NVivo 12 data management software (QSR International Pty Ltd., Version 12, 2019, Victoria, Australia). The Framework Method, used widely across health services research, offers a systematic and flexible model for supporting qualitative thematic analysis [20]. It is particularly useful in situations where there is a desire to identify themes through making comparisons both within and between cases [20]. This was the aim of the current research which set out to explore the experiences of SSAs from the perspective of both parents and health professionals. The framework approach involves a five-stage iterative process of: 1) data familiarisation; 2) identification of a thematic coding framework; 3) indexing and further refinement of the coding framework; 4) charting; and 5) mapping and interpretation to search for patterns and explanations in the data [21]. During the initial data familiarisation stage, members of the FLAMINGO qualitative team (EK, CM, EF, PH) independently read a sample of four transcripts and field notes, two from each participant group (parents and health professionals), in depth and a high-level coding framework was independently devised by each member, based on the study aims and the interview topic guide (S2 and S3), as well as the identification of additional codes identified in the data. The group met on several occasions to discuss overlaps and differences between the four coding indexes which were merged to form the first version of the coding framework. This framework, and the main thematic areas to focus on in the analysis, was then shared with the wider FLAMINGO team for additional comments and input. A subset of six transcripts was independently coded by EK and CM and the results compared to reveal considerable reliability in coding. Subsequently, minor changes to the codes were made, in order to further improve consistency.

The third indexing stage involved systematically applying the analytical coding framework to the remaining transcripts in NVivo. Line by line coding was carried out by three researchers (EK, CM, EF), with ongoing discussion and debate at weekly meetings. A framework matrix was developed to allow for organisation of the data samples (parents and health professionals) by themes to facilitate cross-case and within-sample comparisons. The FLAMINGO qualitative team reviewed the final themes to reach consensus in the mapping and interpretation of the data, thus enhancing rigour and trustworthiness.

### Ethics approval and ethical considerations

The study was approved by the NHS North of Scotland Research Ethics Service (REC reference: 19/NS/0134). Local permissions were also obtained from individual Health Boards.

## Results

### Characteristics of sample

One hundred and nine health professionals were sent an email invitation. Of these, four actively declined and 57 did not reply. The remaining 48 participated in an interview. Of these, three health professionals worked in Health Boards not included as one of the five case sites.

Sixty-four parents contacted the study team in response to study advertisements. Of these, 21 did not meet the eligibility criteria and 22 did not respond to further contact. Twenty-one parents participated in an interview. Six parents were recruited towards the end of the recruitment period when participants were accepted from outside the case areas and came from two non-case study Health Boards (Table 1).

**Table 1:** Characteristics of participants (n = 69).

Parents and children (n = 21)		
Characteristic	Number (n)	Percent (%)
Parent’s relationship to child:		
Mother	20	95
Father	1	5
Gender of child:		
Female	5	24
Male	16	76
Age of child at time of admission:		
0–6 months	4	19
7–12 months	4	19
1–5 years	8	38
6–10 years	4	19
11–16 years	1	5
Reason for admission (infection vs non-infection):		
Infection	15	71
Non-infection	6	29
Time elapsed since child’s admission and the date of interview (months):		
0–6	7	33
7–12	5	24
13–24	5	24
>24	2	10
Missing data	2	10
Admission was pre-COVID (pre-March 2020):		
Yes	7	33
No	14	67
Case Health Boards		
Health Board 1	1	5
Health Board 2	6	29
Health Board 3	3	14
Health Board 4	2	9
Health Board 5	3	14
Other Health Boards	6	29
**Health Professionals (n = 48)**		
**Role and Setting**		
**Primary Care/General Practice/OOH Service**		
GP	10	21
Paediatric Advanced Nurse Practitioner (PANP)	4	8
Advanced Nurse Practitioner (ANP)	1	2
Health Visitor	1	2
**Paediatric Hospital Emergency Department**		
Consultant Paediatrician	7	15
Specialist Registrar in Paediatrics (SpR)	1	2
Specialist Trainee Doctor in Paediatrics (ST)	4	8
Staff Nurse	3	6
Specialist Nurse	3	6
**District General Hospital Emergency Department**		
Consultant Acute/Emergency Medicine	4	8
Senior Charge Nurse	1	2
Paediatric Advanced Nurse Practitioner (PANP)	4	8
Advanced Nurse Practitioner (ANP)	1	2
Specialist Nurse	4	8
Length of experience (years)		
<5	3	6
5–10	4	8
11–20	13	27
21–30	17	35
>30	4	8
Missing data	7	15
Case Health Boards		
Health Board 1	9	19
Health Board 2	9	19
Health Board 3	9	19
Health Board 4	9	19
Health Board 5	9	19
Other Health Boards	3	5

In total, 69 interviews were conducted with health professionals (n = 48) and parents (n = 21) with experience of SSAs. Health professional participants included those working across primary care, district general hospital EDs and children’s hospital PEDs. Almost half (44%) of participants held more than 20 years of experience in their profession. Of the 21 family interviews, 20 were conducted with the child’s mother and one with the father. The age of their child at the time of the SSA ranged from three days to 16 years with 76% being under five years. Whilst all SSAs involved an acute illness, as per the inclusion criteria, 71% of children could be categorised as presenting with an infectious illness. Characteristics of participants are outlined in Table 1.

### Themes

Five themes were identified around the outcomes that are important in existing care pathways of an acutely unwell child between home and a short stay hospital admission. Conducting both within-case and cross-case analysis allowed for the identification of shared themes and outcomes of care that mattered to both parents and health professionals as well as contrasting outcomes that mattered principally to one group and less to the other. Safety of the child was identified by both parents and health professionals as the overarching outcome. Two additional themes identified by both parents and health professionals were reduced uncertainty and anxieties about the illness trajectory and the value of a period of skilled observation. The theme which mattered principally to parents was easy and/or direct access to urgent care, preferably from paediatric specialists. The theme which was mostly unique to health professionals was to reduce hospital admissions.

### Overarching outcome

#### Safety of the child

Promoting the safety of children emerged as an overarching guiding principle and outcome that mattered to everyone involved in the unscheduled care pathway of children presenting with an acute illness. Parents described occasions during which they found it difficult to determine whether their child’s symptoms were indicative of a minor illness or something more serious and thus preferred to “*err on the side of caution*” by attending hospital. Similarly, parents were satisfied when health professionals demonstrated a safe and cautious approach by admitting their child to hospital as explained by one parent:

*“I think had we been sent home that evening I would have been very worried about things I think*, *so I was much happier that they were taking a sort of cautionary approach*.*”*

(Parent 05 HB3)

In the context of preventing SSAs from health professionals’ perspectives, safety always takes precedence. With increasing rates of hospital admissions, a requirement to reduce SSAs, where possible was voiced. The continued challenge, as described by one GP, is maintaining that fine balance between preserving safety and reducing SSAs:

*“It’s really tricky isn’t it*, *the balance of safety versus reducing admissions is the age-old conundrum*.*”*

(HP30 GP HB6)

The diagnostic challenges in differentiating between minor and more serious illness in young children experienced by parents were similarly expressed by most health professional participants, including both referring and receiving clinicians. The uncertainty that is inherent in providing unscheduled care for children was clearly articulated and managing any uncertainty described as being of great importance to reduce risk and preserve safety:

*“Most of these children have mild self-limiting illness but*, *as anybody who works in paediatrics knows*, *it can be difficult to differentiate between a child with something quite mild who presented with a fever to somebody who presents with a fever and actually is quite poorly*.*”*

(HP23 Nurse HB3)

Challenges identifying acute illnesses which were self-limiting and those which were more serious were heightened in health professionals with less experience in paediatrics, further highlighting the importance of prioritising safety and remaining cautious which often resulted in admitting the child to hospital. This was described particularly in relation to infants who make up most attendances in the ED and are a high-risk group, as articulated by one consultant:

*“So*, *you’ll often find that those junior staff will err on the side of caution*, *particularly with certain groups of patients because the other thing we need to look at is the variation in age in our [ED] attendances*. *The majority of our patients are less than a year old*, *and of course if you’re a brand-new paediatric trainee or if you’re an emergency medicine trainee who’s never done paediatrics*, *that’s the group that are going to terrify you the most because they’re most at risk of dying*, *they’re most at risk of sepsis and therefore you’re not going to want to take any chances*…*”*

(HP14 Consultant HB3)

For those working within primary care, dealing with uncertainty was a regular challenge that was widely recognised by paediatric services. In contrast to referrals made to adult services, paediatric services tend to accept referrals without question, thus further acknowledging acceptance of the inherent uncertainty around acute illness trajectory in children, as explained by one GP:

*“When we refer children*, *babies*, *kids*, *whatever it is*, *there’s no debate about the referral*, *they take the referral quite happily*. *I think paediatrics also understand that dealing with uncertainty can be quite challenging*.*”*

(HP04 GP HB3)

At the point of sending a child home from hospital, safety netting is essential so parents understand how to manage the illness at home and know when and where to seek further help should the child’s condition worsen. The quotes below illustrate the importance of safety netting in prioritising safety from the perspective of both a parent and health professional:

*“I was quite worried because we hadn’t got an answer for it*, *but they did the investigations that they could and they did give me open access to the ward*, *so I felt safe enough to come back in or*, *you know*, *so that was pretty good*.*”*

(Parent 12 HB2)

*“We find that safety netting makes a big difference because they [parents] have that security blanket when they leave*, *you know*, *if you’ve got a child that’s been hot and a bit bothered and mum’s worried that they’re intake is not as good*, *they know they can phone up and bring them back and they’re seen*, *whether it’s by an advanced nurse practitioner or a paediatric doctor*.*”*

(HP29 Nurse HB1)

Safety netting is also required within primary care, particularly with short, often ten-minute appointments regardless of the nature of the presenting problem, and with extra urgent demands interceding in busy schedules. GPs assessing an unwell child require assurance that the parent will be able to correctly identify when the situation has escalated and the point at which additional assessment by a health professional should be sought. Without such assurance, GPs are more likely to adopt a cautious approach in accordance with their obligation to protect children and send the child to hospital for further assessment:

*“So*, *you try and get an idea of how stable or safe the home environment is or how…it’s hard to put your finger on it really but if you’re happy with the parent’s ability as well*, *they seem to be understanding what you’re saying on it really*, *they seem to be understanding what you’re saying about worsening signs*. *So they seem to take in the kind of things that you would worry about and why you would want to see the child back*. *If there’s any concern about that it would certainly lower the threshold to ask the hospital to see them*.*”*

(HP09 GP HB4)

### Key outcomes

#### Reduced uncertainty and anxieties

The desire for reassurance from trusted health professionals was a key driver in parents accessing urgent care. Parents explained how they wanted to be reassured that their child’s condition was not serious or life threatening and to be given assurance that they were self-managing the condition in the best way. Observing a child within the ED with additional tests and investigations carried out provided such reassurance and relieved anxiety:

*“Yeah*, *the fact that they had done blood tests and the fact that they wanted to keep [child] in and then they were happy for [child] to go*, *you know*, *that kind of reassured me*. *The fact that*, *you know*, *they said ‘no*, *[child’s] not going home straight away*, *we’re keeping them in’ and then they thought ‘well do you know what*, *[child] seems okay so we can let them go’ so that was reassuring in itself*.*”*

(Parent 21 HB3),

The desire for reassurance was also acknowledged by health professionals as a key motive for parents accessing urgent care. Health professional participants described their experiences of and the challenges around being able to reassure parents that it is safe for them to care for their unwell child at home and that the child does not require care in hospital. In situations where health professionals have not been able to successfully reassure parents, it is more likely the child will be admitted:

*“I think a lot of parents that I see*, *the children are well enough to be at home and they are just really looking for reassurance that their child is not going to die and is going to get better*, *and for a lot of those children if you can confidently say ‘yeah*, *this one’s okay*, *don’t worry about it you’re going to be safe at home’ then that’s kind of acceptable to them*, *and if you’ve not managed to convince them of that it’s much more difficult to get them home*.*”*

(HP01 Doctor HB6)

The reciprocal interaction between clinicians and parents when negotiating anxieties and mutual reassurance about safety in decision making was evident in accounts of referral and admission. Parents explained that having health professionals who take the time to clearly explain their child’s symptoms or diagnoses and the reasons why they recommend discharging the child to home, is effective in providing reassurance:

*“They [Hospital staff] were really*, *really good at explaining it…so I was quite reassured*, *you know*, *they’d explained the reason why they’re letting us go [home] cause it’s really worrying when you leave in case they [child] get worse*.*”*

(Parent 11 HB1)

Parental and professional uncertainty about the illness trajectory and their appropriate anxiety was identified by health professionals as a primary reason for both the referral and admission of a child to hospital, regardless of the pathway that brought them there. As explained by one GP, parental anxiety can influence a GP’s decision to make a referral to hospital even if the GP is not convinced this is a necessary course of action from a clinical perspective. The emotions of parents combined with the fact that they know the child best, can inform a GP’s decision making:

*“Sometimes you refer them up [to hospital] because of parental anxiety…I mean*, *like the hydration one*, *you know*, *their nappies are a bit drier than normal*, *they still look quite well but maybe they’re a bit cool and you know*, *maybe they would be okay but my kind of opinion is better to be safe than sorry if you’re*, *you know*, *cause I know that they can just change so quickly and it’s often the mum or dad that will sway your decision*, *it’s just like ‘they’re not themselves’ you know*, *and once they start saying that you’re probably more likely to refer them…I’m always a bit like ‘I’m not sure if this is really necessary*?*’ and they often do just get seen and discharged*.*”*

(HP31 GP HB6)

Reciprocal anxiety and uncertainty about the illness trajectory influenced decisions to admit a child for a longer period of observation or a short admission as described by two health professionals:

*“With parental anxiety*, *parental concern*, *there’s more of a tendency to keep the child in for a longer period of observation and keep them in overnight*, *even if they are local*.*”*

(HP07 Doctor HB7)

*“It plays a role in my decision making as well because if I have a parent who’s really anxious and doesn’t want to take their child home then I’ll keep them in*, *because I can’t send a mum home who doesn’t want to go home and what if something happens to that child*, *you know*, *so I think it plays a massive part*.*”*

(HP29 Nurse HB1)

Being able to provide reassurance and lessen any anxieties about illness trajectories and adverse outcomes was a desired and shared outcome. However, to achieve this outcome successfully, a referral to hospital and/or a short admission was often required.

#### A period of skilled observation

A consistent theme across the interview data was the value of a period of observation, which may take place in the hospital and which forms a fundamental part of the management plan in children with acute medical illness. Participants explained how being able to observe the child as the trajectory of the acute illness unfolds was of great benefit. Timing of first presentation and clinician seniority can impact on admission decisions:

*“So parents are presenting very early on in the course of their child’s illness*, *often before any kind of focus or infection has become obvious*, *and as a consequence of which you’re left in a situation where again you would be having to make a discharge under quite considerable uncertainty and whilst that might be something that I’m comfortable with doing*, *it’s not something that I would recommend that the very junior staff in my department take a chance with*.*”*

(HP14 Consultant HB3)

Several health professionals described how the passage of time was an essential factor in being able to effectively assess and observe an unwell child before, ideally, being able to send them home safely. The four-hour target, a UK-wide government directive, aims to ensure that the time between a patient arriving in the ED to being either admitted, transferred, or discharged is no longer than four hours. Within the ED setting, there is value in a period of skilled observation of children presenting with an acute illness and, in some cases, admissions might be avoided if health professionals have “*a wee bit longer*” to observe the child and not be restricted by the four-hour UK government ED waiting time target, as described by a Consultant:

*“Quite often the correct and prudent safe management of some presentations that you see in children and young people does involve a period of observation if you’re not able to reach a point at which you’re happy to safely discharge a patient within that four-hour timeframe*.*”*

(HP18 Consultant HB3)

This then leads to a short admission of the child to allow that period of observation to take place:

*“If they’re [children] staying less than 24 hours they’re generally there for observation purposes…so it’s children with common presentations that perhaps just need a longer period of us watching them*.*”*

(HP17 Doctor HB3)

There was congruence between parents and health professionals with respect to the need for and benefits of an extended period of observation, even if that required a short admission to hospital. In the following quote, a parent describes their feelings on the decision to admit their child for a period of observation:

*“I was quite pleased*. *I’d rather be here [hospital] than getting [child] home and worry about it…but they did keep us regularly updated saying ‘this is what our thinking is*, *this is what we’re looking for*, *we’re going to come back and check in an hour or so’ so they kept telling us what they were doing and how often they were going to do it and all that kind of stuff*.*”*

(Parent 05 HB3)

There was some suggestion by participants working within a hospital setting that observation does not necessarily need to be undertaken in hospital, but this currently happens due to lack of appropriate structural capacity for paediatric observation and availability of appropriately skilled staff to perform it in the community:

*“I think because we don’t have the community infrastructures in place to be able to say*, *‘you were utterly distraught at two in the morning*, *we calmed you down*, *you went home*, *and this person is going to come out to your house to see how you’re doing in the day’ because we don’t have that level of sophistication then we often use the hospital as a safety net*.*”*

(HP02 Consultant HB6)

This concept was reiterated and further elaborated on by a number of GPs describing the limitations around time and space to undertake observation of an acutely unwell child within primary care. Many GP practices currently lack the appropriate physical space to be able to observe a child over several hours. There are also constraints around the service opening hours and dependant on what time of day the child presents to a GP practice, there may not be sufficient time to observe the child and therefore referral to hospital is the rational option:

*“We’re quite tight for accommodation*. *If we see somebody in the morning and they’re borderline*, *we’ll sometimes follow them up in the afternoon*, *either face to face or by phone but if it’s somebody we see in the afternoon we don’t have that option I guess and I don’t think we’ve really got the ability to park somebody for a few hours to observe them*.*”*

(HP22 GP HB3)

Having capacity and better infrastructure to assess and observe an acutely unwell child within primary care services might be effective in reducing hospital admissions as suggested by this GP:

*“And could that have been prevented earlier downstream by just a little more attentive assessment in the community*?”

(HP30 GP HB6)

#### Easy and/or direct access to urgent care, preferably from paediatric specialists

Whilst ease in accessing health services is a shared outcome desired by both parents and professionals, interview data suggests that any delay in being able to access urgent care, or experiences in navigating a complex and complicated system, results in parents attending the ED/PED where they have greater confidence that their child will be seen promptly by someone with the appropriate skills. This is articulated in the quotes below from the perspectives of both a parent and hospital consultant:

*“So*, *because it was a Saturday obviously our own GP wasn’t open*, *so the choice is either ED or NHS24 and I thought I’ll phone NHS24 because that’s where we’ve always gone before*. *Phoned them*, *we’ve seen an out of hours GP before*, *they’ve given [child] steroids and [child] has usually been okay*. *But on this occasion when I phoned NHS24 the person I got through to put me onto a nurse practitioner*, *because obviously the call handlers have access to the nurse practitioners in the background*, *and she listened to my son’s breathing and said ‘oh*, *[child] doesn’t sound like they’re in dire distress’ basically ‘you’re just being a panicky mother’ and then said ‘well look*, *it’s your choice if you need to make an appointment with the out of hours GP for four hours’ time’*, *they had a four hour wait at that point*, *‘or obviously if you feel that concerned you would take him to ED yourself’ so that’s what we did*.*”*

(Parent 07 HB9)

*“The usual answers are ‘I can never get through to my GP on the phone’ or ‘I can never get an appointment’ and the same with NHS24*, *‘I tried them once before and it took four hours for them to phone me back therefore*, *I’m never phoning them again and if my child’s unwell I’ll just bring them to the ED’*, *I’ve certainly had that said to me*.*”*

(HP40 Consultant HB1)

Additionally, juggling family demands, employment responsibilities and a hectic lifestyle means that care is often needed outside of primary care hours and the ED/PED is an attractive option as it is always open and accessible. Several health professional participants referred to the accessibility of the ED and the goal of assessing patients within a four-hour window policy has made the ED a “*victim of our own success”*. As explained by one Consultant, unless equally accessible and suitable alternatives to the ED/PED are available, the increase in presentations and admissions will persist:

*“People want their health care*, *and they want it now and that’s unfortunately the way of the world now*, *it’s the sort of Amazon generation…So*, *unless there’s something put in place that’s just as palatable and just as accessible [as the ED/PED] then that’s just going to continue to rise*.*”*

(HP14 Consultant HB3)

In addition to easy and timely access to care, parents often have the perception that the most appropriate professional to assess their acutely unwell child is a paediatric doctor and this can be an additional contributing factor to parents attending hospitals for unscheduled care:

*“I think educating the general population on caring for a sick child and when to seek help because I think we have a society now where people want to see a paediatrician*.*”*

(HP23 Nurse HB2)

Our parent participants outlined several factors which, from their experiences, contributed to them presenting to the ED/PED for their child to be seen. Parents are more likely to seek a second opinion from hospital-based health professionals if they are either not reassured by or in disagreement with the assessment provided by another service providing urgent care, such as GP practices or NHS24. This was discussed by both parents and health professionals in the interviews and is articulated in the quotes below from the perspective of a parent and a primary care ANP:

*“Like I said you’ve always got that fall back where if you’re not happy with what your GP said you can get that second opinion from NHS24*. *I know that isn’t why it was set up*, *you know*, *obviously it was a set up for out of hours when the GPs aren’t open but I think it’s a good thing that if you phone the GP and within a couple of hours things change*, *you’ve got the NHS24 to speak to straight away and then they can refer [child] straight to hospital so I’m quite happy with how you can access that*.*”*

(Parent 21 HB3)

*“Sometimes parents need a bit of reassurance and even though we’ve examined them and not really found anything specifically wrong with them*, *they want a*, *for want of a better word*, *specialist input and they’re usually quite happy to go up to be seen by a paediatrician just to kind of*, *you know*, *reiterate what we’ve done*. *So yeah*, *I think the parents are quite happy to go [to hospital]*.*”*

(HP39 Nurse HB1)

Parents acknowledged the intense and growing demands on the health service and described their ‘duty’ to use services in a responsible way, accessing the most appropriate part of the urgent care pathway for their need at that time. However, a poor experience in any part of the pre-hospital system can result in direct presentation at a hospital subsequently as articulated here:

*“I would definitely listen to my instincts with it*, *and I would still*, *well depending on what happened*, *I would still phone NHS24 for initial advice but if I don’t agree with what they said*, *if I thought anything was seriously wrong*, *I’d just take [child] straight up to the hospital*, *which is what I wish I had done the first time rather than listening to NHS24*.*”*

(Parent 04 HB4)

Parents’ view that hospital-based health professionals are best placed to assess their unwell child contrasts with the perspectives and experiences of health professional participants who, with a wider understanding of the set-up and provision of the health service, accept that the current system does not lend itself to this and only adds to additional pressures managing the patient ‘*flow*’ within the system. The growing trend of wanting their child assessed in hospital poses challenges for the service, as articulated by one doctor:

*“I think the numbers that are coming through in that kind of culture of ‘oh we need to get them checked out’ [in hospital] is probably*, *I don’t have enough experience to say it’s on the up*, *but it feels like it’s worse than when I first started and it’s probably not very sustainable in the current set up*.*”*

(HP01 Doctor HB6)

Differences in health care provision across other countries, particularly within Europe, could in part explain some of these expectations as outlined in the following quote:

*“Patient expectations…again we think maybe particularly so for people who are not used to the health service in this country*, *who do not appreciate the role of general practice and out of hours healthcare*, *expect to see a paediatrician*, *and they don’t think they’ve been given an adequate opinion until they’ve seen a paediatrician*, *and I think that is more so for people who come from outside the UK*. *We’ve certainly seen that*. *So*, *there is an element of parental expectation and how they perceive the health service works*.*”*

(HP05 Consultant HB7)

Interpretation of the interview data suggests that having ease of access to urgent care facilities and a preference for acutely unwell children to be assessed in hospital, preferably by staff with expertise in paediatrics is an outcome valued by parents. Health professionals agree with the requirement to ensure the urgent care pathway is easy to access and appreciate parents’ perception that hospital offers advantages, however, continued increases in use of hospitals in this manner is considered unsustainable in the long term.

#### Reduction in hospital admissions

An outcome that matters principally to health service providers, who have a clearer insight into the pressures on the hospital system, is a requirement to manage hospital flow by providing unscheduled urgent care in an alternative setting whenever safely possible. There was acknowledgement by participants that ED attendance rates and short hospital admissions are continuing to rise and there is now a clear and present requirement to address the resulting capacity issues. The terms ‘inappropriate’ and ‘unnecessary’ were mentioned across some of the health professional interviews to describe SSAs, with clarification that it is not the presentations of many children to hospital that are inappropriate but rather the capacity and system that are not functioning appropriately:

*“So there’s an issue with triaging from NHS24*, *there’s also an issue with people walking into the out of hours centre*, *they don’t phone NHS24 they just come up with their kids*, *but again I think the service in [City] has evolved that way and it’s not really fair to criticise parents for that*, *I think that they just come up when they’re worried and many parents that I meet in general practice are worried appropriately because they’ve tried lots of different measures*, *you know*, *they’ve given Calpol [paracetamol-based medicine for children]*, *they’ve done the usual sort of common sense things and they’re coming up because they just feel the child isn’t picking up*. *So*, *the use of the service isn’t always in the way that it’s designed or appropriate for the way the service thinks it’s being used*, *but it doesn’t mean that the presentations are inappropriate*.*”*

(C004 GP HB3)

Whilst reducing hospital admissions was a preferred outcome for health professionals, there was consensus across those interviewed that it is not helpful to deem admissions as ‘*inappropriate*’ since such terminology could dissuade families from attending ED and have serious resultant implications:

*“I suspect there isn’t any inappropriate ones*, *like I say*, *if you’ve got a concern at the time then it’s always appropriate*, *I would say*.*”*

(HP09 GP HB4)

*“I think in my view what is appropriate/inappropriate for us as health professionals is completely different when it comes to parents*, *and I think the issue here is you need to be careful in my opinion of what you define appropriate and not appropriate because what we feel is trivial from a clinical perspective parents might feel no*, *actually this is really essential*. *So*, *it is a difficult concept*.*”*

(HP10 Consultant HB4)

Instead, health professional participants suggested it is important to consider the concerns that brought families to the ED in the first place and to direct them to alternative health services that are best suited to their child’s care needs. They acknowledged a range of factors contributing to ‘*inappropriate*’ hospital attendance which included difficulty accessing a GP appointment; having to wait a lengthy period when contacting NHS24; reduced confidence in some health professionals following a poor previous experience; lack of awareness of the most appropriate service to provide urgent care; or the search for a second opinion.

When exploring alternative care pathways for children who are acutely unwell, one consultant suggested this be undertaken in a person-centred way and in partnership with families:

“*You need to look at the routes*, *what’s made them suddenly at six o’clock come to A&E*, *you know*, *what is the system*? *Where is the fault here*? *Is it they [parents] can’t get an appointment with the GP*, *is it they don’t trust their GP*, *is it because it’s too difficult to*, *so this is where I think we need to find with the people a solution to assist them so people end up in the hospital only when needed*.*”*

(HP10 Consultant HB4)

Whilst safely reducing hospital admissions was a key outcome primarily for health professionals, parents, when asked if they perceive short admissions as being necessary, were aware of the pressures facing the health care system and acknowledged the need to not over burden the health service:

*“As I said I can’t fault them*, *for the pressure that they’re under they always*, *the service is always so high…I can’t thank them enough for what they do for my kids*, *so I’ve never complained about the NHS*, *they’re always so friendly regardless of how much pressure*, *how busy they are*, *they always give you the service that exceeds anything*.*”*

(Parent 01 HB3)

Despite this, parents, when reflecting on their experiences of short or zero-day admissions, deemed them to be necessary:

*“In my experience*, *I’d say they’ve [short admissions] always been necessary for my child*. *I don’t know about others*, *but yeah*, *I’d say they were necessary because if they had sent my child home and obviously the worst had happened then obviously it would have been wrong*, *but they made sure [child] was fine and then sent them home*.*”*

(Parent 12 HB2)

*“I wouldn’t say anything’s really unnecessary if it’s going to get your child looked over by a professional*, *that’s going to advise you on what’s happened and the chances of this happening again and what to do*. *I think if I hadn’t went [to the ED] I would have probably started worrying what happened if it is something else and I don’t know*? *But I think to get that reassurance I wouldn’t say it was…it’s an inconvenience yes because you’re up during the night*, *you’re exhausted then you miss school the next day if you’ve got other kids*, *but I don’t think it’s wasted at all*.*”*

(Parent 11 HB1)

## Discussion

This paper aimed to provide an in-depth account of the experiences of SSAs from the perspective of parents accessing unscheduled urgent care for their acutely unwell child and of health professionals referring, caring for, or admitting children for SSAs. Parents and health professionals, in describing their distinct experiences of accessing or providing urgent care for acutely unwell children that often led to short hospital admissions, identified both shared and unshared outcomes of care that matter. Acknowledging that the rising rates of SSAs are unsustainable within current hospital paediatric services, there is a fundamental need to identify ways to modify existing urgent care pathways. Our findings identify that shared outcomes of care that are aligned with stakeholder needs will be critical to the success of any change to existing care pathways and need to be considered by healthcare policy makers and planners tasked with pathway redesign.

Preserving safety was paramount and a shared overarching outcome for all, driving actions, behaviours, and experiences. Parents’ and professionals’ uncertainty, anxieties and challenges in determining whether a child will transpire to have a minor or more serious illness was the driving factor in taking a cautious approach to both attend and be admitted to hospital. This was particularly the case for those professionals with less experience in paediatrics, worried and concerned parents and those with previous unsatisfactory experiences of care. These findings corroborate those of a smaller Scottish study conducted in a single site similarly interested in factors influencing the decision-making around emergency admissions of children [22]. Interviews with parents revealed that adopting a ‘better safe than sorry’ approach supported their decision to attend hospital if they were unsure about their child’s illness. Likewise, referring and receiving clinicians reported that ‘erring on the side of caution’ was a driver of admissions [22]. Our findings also concur with previous qualitative interviews with parents suggesting that ‘averting risk’ and ‘avoiding potential harm’ were key drivers in parents’ decision making to access unscheduled care for their acutely unwell child in Ireland, a theme which resonated strongly in our findings [13].

Another strong shared theme was the value of having both the time and appropriate physical space to undertake a period of skilled assessment and observation to determine the child’s illness trajectory, make safe decisions and reassure all involved. This concurs with a study examining admission of adult patients to hospital from the ED, suggesting admissions may be safely avoided if hospital staff had additional time to observe and manage patients [23]. The national four-hour ED waiting target in the UK may be one of the factors driving admissions given that health professionals in ED may feel that observation for an additional hour or two beyond this window is required before being confident that they can be sent home, a point emphasised by several of our participants. However, trends in SSAs do not illustrate a notable increase in or around 2004 [1] when this policy came into place suggesting the situation is complex with many factors involved.

Acknowledging the value of a period of skilled observation raises the question of whether this needs to be carried out in hospital or could take place in an alternative community setting. Evidence from our study and other published work suggests that parents attend the PED/ED as other parts of the urgent care pathway are too complex to navigate, difficult to access, and overly time-consuming [9, 13, 24]. Watson and Blair [9] concur with this, proposing that the PED has become an attractive option for parents of children with non-urgent illnesses and a ‘good’ first experience then initiates a parental expectation that safety equates to being seen by a paediatrician in an ED or hospital and is likely to be a key determinant of their future behaviour. This leads to a growing trend of parents wanting their child to be assessed in hospital, as suggested by participants in our study, and additionally the four-hour waiting target promises a paediatric opinion within a short timeframe.

Being reassured that an illness is self-limiting and that an appropriate management plan is in place for safe care at home, was a shared outcome that mattered to parents and health professionals, with consequent relief of any anxiety and uncertainty. Parents’ need for reassurance is widely documented in the literature [13, 25, 26] including a systematic review [24] on factors influencing parental preferences and decision making when seeking unscheduled health care for their children. De Bont and colleagues [25] interviewed parents presenting to GP out-of-hours care with a febrile child and described an interesting discrepancy where parents’ fears and anxieties were not based on their knowledge of symptoms but on their emotions at the time. Heightened emotions can often be the driver in accessing urgent care, and influence when and which part of the health service parents present at, with the GP undertaking a physical examination of their child succeeding in reducing their anxiety [25]. This concurs with our findings that parents will seek a trusted professional who can relieve their worries. Importantly at the initial point of contact with the health service, parents want to feel confident and reassured that the professional who assesses their child has skills in acute paediatrics, regardless of discipline or seniority, and will facilitate timely, efficient and safe management of the situation. Our data revealed a range of scenarios where out of hours GP services, ED or hospitals could all be perceived as either meeting or not meeting this requirement.

The language used in reference to reducing ED attendance and hospital admissions, both in adult and paediatric populations, is often centred around them being either ‘inappropriate’ or ‘unnecessary’. This is a trend observed both in the UK and in several other high-income countries [13, 27–30]. Within our study, there was strong agreement amongst health professionals that, whilst there is a requirement to reduce short stay admissions, such terminology is not helpful and could compromise safety if parents are discouraged from attending. Moreover, the view that an admission could have been avoided negates an in-depth understanding of the home, community, or the primary care context. Whilst, in an ideal situation, an admission may be avoided, this is not always possible in the real world of competing demands on time, place and person.

Reducing SSAs was not an outcome desired by parents, who instead prioritised the benefits of having their child assessed in hospital, signifying that this should not be the driver when considering interventions aimed at improving urgent care pathways for children. Shared outcomes that matter are more likely to be successful drivers of change. The challenge is to identify what an effective change looks like. A recent systematic review of interventions to reduce unscheduled admissions of children to hospital concluded there is lack of robust evidence to support their effectiveness and, interestingly, few included interventions focused on the pre-hospital pathway [31]. Conversely, interventions targeted at the pre-hospital pathway showed potential to reduce ED attendance largely amongst the adult population, whilst equally lacking sufficient quality evidence to make conclusions around their effectiveness [32]. This adds further impetus to the requirement to first prioritise and then develop interventions through partnership between parents, health professionals, policymakers and planners, to address the ubiquitous and unabating problem of rising rates of SSAs. Importantly, as our findings have indicated, outcomes of care that matter to parents and health professionals should be at the centre of this process. This could be ensured by embracing co-design approaches to change urgent care pathways.

### Strengths and limitations

This paper offers valuable insight into parents’ and health professionals’ perspectives on the urgent care pathway from home to hospital for acutely unwell children who experienced short stay hospital admissions. The rigorous qualitative approach of working with the multi-disciplinary FLAMINGO team of researchers, clinicians, and PPI representation with topic experience is a key strength. The FLAMINGO study is unique in firstly reporting results of data-linkage on SSAs [15] and then on the experiences of unscheduled paediatric care of both parents and relevant health professionals to understand the meaning behind the numbers. Undertaking a mixed methods approach offered numerous benefits and contributed to the overall rigour of this study. For example, early quantitative analysis of the linked data identified trends in SSAs across the country and these figures informed selection of case sites to ensure differing admission rates.

There are some limitations to acknowledge. The COVID-19 pandemic had considerable impact on our recruitment strategy, especially for our parent sample. Restrictions meant we had to recruit remotely instead of through the planned presence of researchers in hospitals. Self-selection may have resulted in the inclusion of parents who experienced a ‘significant’ SSA, or had a particularly positive or negative experience, rather than those with more typical admissions. Despite being a convenience and self-selected sample, over 75% of the parents we interviewed had a child under five years of age and were seeking urgent care for an infection which concurs with published evidence on key characteristics of unscheduled hospital admissions in children [1, 3, 5, 33]. The challenges posed by the pandemic did not impact the project’s considered sample size of 72. The sample of health professionals included representation from most services providing unscheduled urgent medical care apart from NHS24 and the Scottish Ambulance Service and represents the diversity of urban and remote acute paediatric structures and acute care pathways in Scotland, some of which are likely to be relevant to the wider UK and countries with publicly funded health services. A potential limitation is that the data are largely confined to mothers, and whilst the interview discussions with mothers were situated around the wider family’s experience, parent/s, carer/s and child/ren, fathers’ perspective may therefore not be fully taken into account. Further research including the unique perspectives of fathers would be of value.

The landscape around accessing urgent care and the flow within hospitals changed significantly during the pandemic, being much quieter and not characteristic of the rising trend in admission rates driving this research [10]. Fourteen of the 21 parents (67%) interviewed focused on a short hospital admission that took place during the pandemic. This limitation was minimised for health professional participants by asking them to discuss pre-COVID-19 experiences first before questions around the impact of the pandemic. Data saturation was reached with common or shared themes identified through our interpretation of the data. We do acknowledge that a wider study population may have provided additional insights.

There is a potential for recall bias amongst parent participants given the time elapsed between the admission experience and interview with parents’ recollections of the event having diminished over time. Finally, our study design focused on the perspectives of parent and health professionals with experiences of SSAs meaning that we did not have opportunity to explore experiences where an admission was avoided. There is potentially some benefit to be gained from evaluating what worked well and what did not in such situations, and this is therefore recommended as a future research focus.

## Conclusions

The continued rise in ED attendance and short hospital admissions underlines the importance of examining both parent and professional experiences of urgent care pathways for children with acute illness to drive improvement. The findings of this study revealed that many outcomes of urgent care that matter to families are consistent with those of health professionals providing care, and, that preserving safety is of paramount importance. Importantly, the outcome of reducing SSAs which the FLAMINGO study set out to explore was not an outcome that mattered to parents as much as it did to professionals. The shared outcomes that mattered to both were protected and adequate time to assess an illness trajectory without time pressures to relieve anxiety, uncertainty and understand the family context, a suitable dedicated physical space to assess and monitor the child, and availability of appropriately skilled professionals in child health. Furthermore, the observed commonality in outcomes between families and health professionals suggests that a partnership approach in prioritising, developing and testing interventions to improve the efficiency, effectiveness and cost effectiveness of urgent care pathways is recommended. The next phase of the FLAMINGO project will bring together mixed methods analysis and explore a range of solutions that meet the aligned values and outcomes that matter to both families and staff. This commences the intervention development process prior to feasibility and effectiveness testing and follows current guidance on approaches and reporting [34, 35].

## Supporting information

S1 File COREQ reporting checklist. (DOCX)

S2 File Semi-structured interview guide (health professionals). (DOC)

S3 File Semi-structured interview guide (parents). (DOC)
