# Supplementary material for: Short stay hospital admissions for an acutely unwell child: A qualitative study of outcomes that matter to parents and professionals
Source: PLoS One. 2022 Dec 16;17(12):e0278777. doi: 10.1371/journal.pone.0278777 (PMC9757586; doi:10.1371/journal.pone.0278777)
Supplement: S1 File — (DOCX) [file pone.0278777.s001.docx]

**COREQ (COnsolidated criteria for REporting Qualitative Research) Checklist**

| **Topic** | **Item No.** | **Description** | **Reported on Page No.** |
| --- | --- | --- | --- |
| **Domain 1: Research team and reflexivity** |  |  |  |
| ***Personal Characteristics*** |  |  |  |
| Interviewer/Facilitator | 1 | Which author/s conducted the interview or focus group? | 6 |
| Credentials | 2 | What were the researcher’s credentials? | 1 |
| Occupation | 3 | What was their occupation at the time of study? | 1 |
| Gender | 4 | Was the researcher male or female? | 6 |
| Experience and training | 5 | What experience or training did the research have? | 6 |
| ***Relationship with participants*** |  |  |  |
| Relationship established | 6 | Was a relationship established prior to study commencement? | 4-5 |
| Participant knowledge of the interviewer | 7 | What did the participants know about the researcher? Eg. Personal goals, reasons for doing the research | 4-5 |
| Interviewer characteristics | 8 | What characteristics were reported about the interviewer/facilitator? Eg bias, assumptions, reasons and interests in the research topic | 4-5 |
| **Domain 2: Study design** |  |  |  |
| ***Theoretical framework*** |  |  |  |
| Methodological orientation and theory | 9 | What methodological orientation was stated to underpin the study? Eg. Grounded theory, discourse analysis, ethnography, phenomenology, content analysis | 4 |
| ***Participant selection*** |  |  |  |
| Sampling | 10 | How were the participants selected? Eg. Purposive, convenience, consecutive, snowball | 4-5 |
| Method of approach | 11 | How were participants approached? Eg. Face-to-face, telephone, mail, email | 4-5 |
| Sample size | 12 | How many participants were in the study? | 7-9 |
| Non-participation | 13 | How many people refused to participate or dropped out? Reasons? | 7-9 |
| ***Setting*** |  |  |  |
| Setting of data collection | 14 | Where was the data collected? Eg. Home, clinic, workplace | 6 |
| Presence of non-participants | 15 | Was anyone else present besides the participants and researchers? | 6 |
| Description of sample | 16 | What are the important characteristics of the sample? Eg. Demographic data, date | 8-9 |
| ***Data collection*** |  |  |  |
| Interview guide | 17 | Were questions, prompts, guides provided by the authors? Was it pilot tested? | S2 and S3 |
| Repeat interviews | 18 | Were field notes made during and/or after the interview or focus group? | 6 |
| Audio/visual recording | 19 | Did the research use audio or visual recording to collect the data? | 6 |
| Field notes | 20 | Were field notes made during and/or after the interview or focus group? | 6 |
| Duration | 21 | What was the duration of the interviews or focus groups? | 6 |
| Data saturation | 22 | Was data saturation discussed? | No |
| Transcripts returned | 23 | Were transcripts returned to participants for comments and/or correction? | No |
| **Domain 3: Analysis and findings** |  |  |  |
| ***Data analysis*** |  |  |  |
| Number of data coders | 24 | How many data coders coded the data? | 6-7 |
| Description of the coding tree | 25 | Did authors provide a description of the coding tree? | 6-7 |
| Derivation of themes | 26 | Were themes identified in advance or derived from the data? | 6-7 |
| Software | 27 | What software, if applicable, was used to manage the data? | 6-7 |
| Participant checking | 28 | Did participants provide feedback on the findings? | 6-7 |
| ***Reporting*** |  |  |  |
| Quotations presented | 29 | Were participant quotations presented to illustrate the themes/findings? Was each quotation identified? Eg. Participant number | 9-20 |
| Data and findings consistent | 30 | Was there consistency between the data presented and the findings? | 9-20 |
| Clarity of major themes | 31 | Were major themes clearly presented in the findings? | 9-20 |
| Clarity of minor themes | 32 | Is there a description of diverse cases or discussion of minor themes? | 9-20 |

Developed from: Tong A, Sainsbury P, Craig J. Consolidated criteria for reporting qualitative research (COREQ): a 32-item checklist for interviews and focus groups. *International Journal for Quality in Health Care*. 2007. Volume 19(6): 349-357.
